# Supplementary material for: Morphological disparity across the K-Pg boundary in mollusk shells: A theoretical morphology approach
Source: PLoS One. 2026 Feb 20;21(2):e0343127. doi: 10.1371/journal.pone.0343127 (PMC12923056; doi:10.1371/journal.pone.0343127)
Supplement: Attachment — Submitted filename: Considerations Rev..pdf [file pone.0343127.s002.pdf]

I sincerely appreciate the opportunity to review this manuscript, which I consider extremely important for the advancement of fossil morphometric studies. The topic addressed is relevant, and the data presented have great potential to contribute significantly to the field.

Overall, the work presents a solid and representative database. However, to achieve this, some aspects need further development, particularly in the Discussion section. When reading an article, the reader expects the results obtained to be contextualized and compared with the existing literature. In this regard, the current manuscript could be enriched. The data appear to be well represented, but the discussion needs to be better developed to go beyond the presentation of the results, integrating them into the already established scientific dialogue.

Another point that deserves attention is the extrapolation of interpretations. In certain sections, local or regional interpretations are replicated to explain global-scale events without further exploring the specificities of the local data themselves. Focusing more on the local implications of the results, before broadening the scope, could strengthen the study's argument.

Transparency and reproducibility are crucial to methodology, so the number of samples (n) for each temporal stratum and each location analyzed must be clearly stated. Lack of this information can introduce bias in the interpretation of the results and is crucial for it to be clearly presented so that the scientific community can accurately assess the robustness of the conclusions. I made specific comments throughout the manuscript, suggesting adjustments and refinements to the ideas presented, if the authors deem them pertinent.

This is a very important and relevant article for fossil morphometrics. I recommend its publication after reviewing the suggested modifications. I am very excited about the potential of this work and confident that, with the adjustments, it will become a valuable reference. I congratulate the authors for their effort and wish them excellent work in the final revision phase.

Introduction:

Line 34: "morphological studies focused on quantifying the diversity of mollusk shell forms" Could you add any studies that have this type of study?

Line 40: The authors state that "Various aspects of morphological variability across geological time can be captured by employing diverse disparity measures," but they do not

exemplify the changes over geological time. Adding an example in this introductory section would make the ideas clearer.

Lines 48-49: In a very general way, it is mentioned that there are studies with morphospatial approaches in mollusks. However, the authors can provide some examples for this text.

Lines 56-60: Taphonomy and diagenesis are absent here; both drastically modify shell morphology, and they cannot be ignored when designing morphospaces. Could you talk about them? Because they directly impact your results.

Line 62: I understand the "biological perspective" of the extinctions. However the geological perspective can not be just ignorated. Macroevolutionary processes are rarely recognized in the fossil record (small fossil data, taphonomy modifications, taxonomic issues, etc.), and the geological perspective is often mapped only by geochemical data. This is important to be mentioned.

Lines 69-71: In fact, paleoenvironments were already being modified throughout the Cretaceous with climate change and faunal turnover. In fact, the Chicxulub impact collapsed niches very quickly, but the collapse of primary production and ocean acidification occurred heterogeneously across the planet. I suggest adding this information so the reader can see the text more clearly.

Lines 69-71 and **75-77:** Reference?

Line 81: The "the most studied macroevolutionary events in the history of life on Earth" is too much. If we are to judge, for life on the planet the Ediacaran-Fortunian boundary was more important.

Line 87: Why were only these classes "Bivalvia and Gastropoda" studied? Any specific reason?

Lines 90-96: This information should be in the paper's Discussion.

Figure 1: I suggest including the number of species, genera, and families in each geological age, or presenting the data in a table. A richness scale of 0–800 is too big and difficult to visualize.

Methods:

Lines 107-110: What was the selection criterion for choosing the families? And why was only one specimen per species studied? Don't you think this might affect your results? Were

they all juveniles? Adults? What was the criterion for choosing the specimens? Please provide clear methodological information.

Line 117: In the "Measures and Theoretical Morphospace" section, I suggest adding a paragraph presenting the ontogeny of these bivalve and gastropod mollusks. Throughout the growth process of both, the morphology of the shells changes. Indicating which ontogenetic phase you are working with is important, thus reducing potential data bias.

Lines 144-153: Reference?

Line 164: In the "Disparity measurements" section. Were normality tests performed on the data? Are they applicable in this case?

Results:

Line 191: In "However, upon closer examination according to the ecological category" when was this "closer examination" performed, was any other test applied? It's important to clarify. Perhaps a Cluster Analysis of the results can help you see how the data clusters over time.

Lines 195-197: Indeed, for a taxon to persist for 10 million years is a long time, especially under oscillatory paleoenvironmental conditions, such as those at the K-Pg boundary. These paleoecological recoveries may also be a faunal turnover; considering the morphometric replacement of the niches presented here, I think it is important to mention or suggest this possibility.

Line 206, 210, 212, 215, 256: Cite the "species richness" here as well.

Lines 209-210: "Immediate" recovery in the fossil/geological record is very difficult to achieve. If we observe Figure 6, ignoring time 3, a decreasing trend (6-c) of Stationary epifauna and Facultatively mobile semi-infauna is notable; however, Facultatively mobile infauna are maintained.

Lines 209-210: "Immediate" recovery in the fossil/geological record is very difficult to achieve. If we look at Figure 6, ignoring time 3, we can see a decreasing trend (6-c) in Stationary epifauna and Facultatively mobile semi-infauna; however, Facultatively mobile infauna show maintenance. If the K-Pg boundary is indeed "the most studied macroevolutionary event in the history of life on Earth," it would have brutally decimated all taxa, impacting all morphospaces. Sometimes the words used in the paper overstate the facts without any cohesive data.

Lines 220-221: Why is the family Mactridae important? References are needed to further support this statement. Is there a reason for the absence of this family in the Paleocene? Taphonomic?

Line 227-231: In fact, semi-infaunal facultatively mobile species failed to recover their morphological disparity; quite the opposite. Figure 6 shows a broadening of their morphometric spectrum, in addition to increasing the number of families from one to three. Could this change be a faunal turnover? How did semi-infaunal facultatively mobile species achieve such success in the K-Pg? Could this extinction be distinct for different taxa?

Lines 234-239: The "Facultatively mobile deep infauna" exhibited paleoecological behaviors similar to those of semi-infaunal facultatively mobile species, increasing their diversity and expanding morphospaces. I would like to see an in-depth discussion, considering the forms and other modifications brought about in K-Pg.

Line 244: Could these disparate values be due to taphonomic and diagenetic factors? If not, how were they differentiated?

Line 274: What is the number of species?

Line 285-286: Suggesting that "these organisms can maintain their morphological diversity through extinction events" without other data is a bit hasty. Is there any other information or comparison that this has occurred over geological time?

Discussion:

Lina 291: "especially when compared to the prevalence of studies utilizing PCA morphospaces" without presenting and comparing the data is irrelevant. I suggest using the data to demonstrate the weaknesses of the PCA test. Apply PCA tests to your data to show and compare test problems.

Line 296: Was the extinction event at the K-Pg boundary homogeneous and synchronous worldwide? If so, focus on your area of study and discuss it briefly. If not, discuss how it occurred differently in different locations around the world. This would make your discussion more interesting for the reader.

Lines 309-312: This paragraph seems more like a result.

Lines 317-318: "Further exploration of proper parameter detection could contribute to the development of new ecomorphospaces for Helicoida", please clarify the reason that prevented you from carrying out this “exploration of proper parameter detection”. I understand that ecology has a direct impact, but it needs to be better supported.

Lines 335-337: Result?

Lines 357-359: In fact, as a reader, the results and discussion present only information/data from morphospaces. I suggest that you develop an in-depth discussion, including paleoenvironmental changes and sedimentary processes, as they directly impact the ecology of animals.

Lines 388-389: The "varied tectonic activity due to ongoing subduction" hadn't been mentioned before. Are they relevant to your results? If so, it's important to discuss this throughout the discussion, not just here. Be careful with "generally maintained an oceanic aspect" as you previously stated in line 367 the "Acidification and suppression of primary productivity in oceans". Make it clear what type of oceanic maintenance you're referring to.

Lines 396-397: The authors said "the broad geographic distribution of the fossils studied here", but they did not clearly present how many were studied from each locality, age, each genus, species, etc. This data is in the supplementary table, but I suggest presenting another reduced table in the results section to facilitate visualization of the data.
