# Supplementary material for: Morphological disparity across the K-Pg boundary in mollusk shells: A theoretical morphology approach
Source: PLoS One. 2026 Feb 20;21(2):e0343127. doi: 10.1371/journal.pone.0343127 (PMC12923056; doi:10.1371/journal.pone.0343127)
Supplement: Attachment — Submitted filename: Response_to_Reviewers.docx [file pone.0343127.s004.docx]

**RESPONSE TO THE REVIEWERS**

Manuscript: **PONE-D-25-43258**

Morphological disparity across the K-Pg boundary in mollusk shells: a theoretical morphology approach

First, we would like to thank the reviewers for their comments, which helped to improve the presentation of our work.

**Reviewers’ comments are in blue italics, authors’ replies are in plain text, and changes in the manuscript considering the recommendations are underlined. In the revised version of the manuscript, the changes are highlighted in blue.**

**REVIEWER #1**

*1. If possible, provide better-resolution pictures of the samples depicted in Figure 3f and 3h (Caryocorbula dickersoni — LACMIP 16115 Type 6592; Corbulidae and Goniocheila wilsoni — LACMIP 17535-6; Aporrhaidae, respectively).*

**Authors’ reply:**
Thank you for this suggestion. Unfortunately, higher-resolution images of these specimens are not feasible because the shells are very small (less than 1 cm), and the current photographs represent the maximum resolution achievable.

*2. Check figures 6 and 9 and/or their captions, which say “a) before the mass extinction (end of the late Cretaceous and b) after this event (Paleogene)”, it seems to be inverted.*

**Authors’ reply:**
We have made the suggested changes in the captions of Figures 6 and 9. We appreciate this observation.

**CHANGES IN MANUSCRIPT:**
The captions of Figures 6 and 9 have been edited accordingly.

*3. In line 209, authors state that intraspecific variation is anticipated to be minimal in the context of shape differences across families and classes, but what exactly supports this claim? Couldn’t some species display significant variations when in different microenvironments or even between individuals of different ages? Perhaps add a quantitative source or a short justification.*

**Authors’ reply:**
We have now included a brief justification in the Methods Section, emphasizing three key points:

Variation. Although intraspecific variation is relevant for understanding morphological diversity, it is generally lower than the variation observed among species, genera, and families.

Model limitation. The model used in this study was designed to capture macroevolutionary patterns in general shell morphology; therefore, small intraspecific differences may not be reliably detected.

Macroevolutionary focus. Whenever possible, we selected specimens from type collections (holotypes, paratypes, or hypotypes) to ensure accurate representation of each species.

**CHANGES IN MANUSCRIPT:**
A new paragraph has been added in line 109 (Methods Section in *Fossil Material* subsection).

*4. In line 236, authors mention that the increase in number of Lucinidae species does not necessarily translate into a greater extension in the morphospace, however, by looking at the graph, it seems it does, slightly*.

**Authors’ reply:**
Yes, that is correct. Although both families share a similar shell morphology with respect to the parameters *k* and *ϕ*, Lucinidae occupies a slightly greater extension of the morphospace. We appreciate your observation.

**CHANGES IN MANUSCRIPT:**
A short clarification has been added to the text between lines 236 and 237.

*5. I must admit that I am having a bit of a hard time observing how Mytilidae occupies the morphospace region previously held by Parallelodontidae (l. 363; Fig. 6).*

**Authors’ reply:**
We appreciate your careful observations. This confusion was due to the imprecise wording of the text. The correct family is *Inoceramidae*, not *Parallelodontidae.* Our intention is to indicate that although some families occupy morphospace regions previously held by extinct groups, Mytilidae preserves its overall distribution within the morphospace despite the extinction of families such as Inoceramidae.

**CHANGES IN MANUSCRIPT:**
The family name has been corrected in line 365, and the text has been clarified.

*6. Line 259, the figure to be cited was indeed 8c, or should it be 8b?*

**Authors’ reply:**
Yes, to illustrate the differences graphically in morphospace, the correct Figure is Figure 8b. We appreciate this observation.

**CHANGES IN MANUSCRIPT:**
The figure citation has been corrected to Figure 8b in line 259.

*7. Line 266, does the creeping semi-infaunal gastropods display higher diversity of morphology during the Cretaceous period than the creeping epifaunal?*

**Authors’ reply:**
If we consider the total area or extent occupied in morphospace, creeping semi-infaunal gastropods do not exhibit higher morphological diversity than creeping epifaunal gastropods. However, the Mean Pairwise Distance (MPD) values for creeping semi-infaunal gastropods are slightly higher in the latest Cretaceous (Time 2 in Fig. 8c), indicating that species within this category are somewhat more divergent from each other on average. In contrast, the Convex Hull area, which reflects the total morphospace extent, shows that creeping epifaunal gastropods occupy a larger area and therefore display greater overall morphological diversity.

To avoid confusion, we have revised the corresponding sentence in the manuscript and added a brief clarification of the difference between these two metrics in line 265 of the revised manuscript.

**CHANGES IN MANUSCRIPT:**
To clarify, we have checked the relevant sentence and added a brief explanation of the difference between these two metrics in line 265 of the revised manuscript.

*8. Lines 281-286, the figures to be cited here might be 8 and 9 instead of 7 and 8.*

**Authors’ reply:**
We appreciate your observation.

**CHANGES IN MANUSCRIPT:**
We have corrected the citations of Figures 8 and 9 in lines 281–286 in the revised manuscript to reflect this.

*9. In a more general note, authors briefly remarked that certain morphological features cannot be fully replicated due to the limitations of the geometric model. Maybe add a small discussion on what this could entail – how, specifically, may it bias the interpretations made from the obtained results? Which ecological signals might be under-represented?*

**Authors’ reply:**
We thank the reviewer for this valuable comment. We briefly mentioned this issue in lines 320–323, but we have now expanded the discussion to address the specific morphological features that cannot be fully replicated by the geometric model and how this may bias the ecological interpretations. In particular, features such as aperture shape and shell ornamentation provide important ecological information, including predator–prey interactions and burial-influencing factors.

**CHANGES IN MANUSCRIPT:**
A brief discussion addressing these points has been added to the last paragraph of the “Geometric parameters” subsection (line 321).

*10. Are all the species in Table S1 indeed presented with their most recent accepted names?*

**Authors’ reply:**
We have rechecked the list of species names in Table S1, and all of them are correct. These names correspond to those used in the collection and reflect the most recent taxonomic updates at the genus and species levels.

*11. Proofread entire manuscript for very few typos and grammar issues. The same goes for the formatting of citing figures within the text.*

**Authors’ reply:**
We appreciate your observation. We have reviewed the text for typographical and grammatical errors, as well as formatting of figure citations.

**REVIEWER #2**

**Major comments:**

1. Sample size and the need for appropriate corrections
Sample size is critical for evaluating the disparity. As the size of the analyzed dataset increases, disparity metrics tend to increase accordingly. Corrections that account for sampling completeness are needed. Moreover, in Time 3, sample sizes for several life modes are reduced to 1-2 specimens, making the current evaluations among life modes fundamentally problematic. Additionally, whether the observed decrease and recovery in disparity represent meaningful changes cannot be assessed without removing the effects of taxonomic diversity decline and recovery. In other words, the results may indirectly reflect changes in taxonomic diversity through disparity metrics, rather than capturing true changes in disparity.

**Authors’ reply:**
We agree that the number of specimens can affect the magnitude of disparity, as larger sample sizes may result in a greater occupied morphospace. However, our study aimed to provide an exploratory analysis of ecomorphological patterns across the K–Pg boundary rather than a statistical comparison among time intervals. We used only well-preserved specimens representing distinct species from standardized stratigraphic collections, ensuring that each taxon was counted once within its corresponding time bin and ecological categories.

We acknowledge that the disparity values for Time 3 are based on reduced sample sizes for certain ecological modes, which limits the robustness of these comparisons. Nevertheless, these reduced values accurately reflect the documented low faunal diversity immediately after the extinction event. Given this limitation, we qualitatively interpreted disparity changes and focused on relative patterns rather than absolute magnitudes.

**CHANGES IN MANUSCRIPT:**
In the Methods section, we have included a brief justification, emphasizing how we selected the families and determined the number of specimens.

2. The theoretical morphological model parameters considered are likely insufficient
Of the six parameters in the theoretical morphological model (Contreras-Figueroa and Aragón 2023. Diversity) used in this study, only two parameters each are considered for bivalves (k, φ) and gastropods (z, a). While I agree that these two parameters for each group are important, as explained in Table 1, the exclusion of other parameters has not been adequately justified. Additional parameters are clearly utilized to reproduce the diverse morphologies shown in Figure 3. Furthermore, previous studies have demonstrated that factors corresponding to other parameters are important for understanding morphological diversity and functional constraints related to modes of life (e.g.,aperture inclination for gastropods; Raup 1966 Journal of Paleontology, Linsley 1977 Paleobiology, Noshita et al. 2012 Paleobiology, Okajima and Chiba 2013 Evolution). If only this subspace of the morphospace is to be considered, it should be explicitly stated that they are examining changes in specific aspects of disparity rather than disparity in general, and the discussion should be limited accordingly (see also Major comment 4 below).

**Authors’ reply:**
It is true that reconstructing a complete three-dimensional model requires all six parameters of the proposed model. However, using all six parameters would necessitate creating a six-dimensional morphospace, which is not visualizable. Principal component analysis (PCA) was performed, and we conducted such an analysis in addition to this work, but it did not yield a clear differentiation of which combination of parameters best represented each shell group. Furthermore, the statistical morphospaces of this type rely on dimensionality reduction methods, producing abstract axes that can be difficult to interpret. In contrast, the theoretical morphospaces used in this study offer the advantage of clearly visualizing how morphology varies along the axes. Therefore, we chose to conduct a literature review to provide an ecomorphological basis for selecting the parameters, as shown in Table 1.

Thanks to your observation, we agree that it should be explicitly stated that the analysis focuses on changes in specific aspects of disparity rather than the overall disparity of shell shape. Accordingly, we have adjusted the discussion to reflect this.

**CHANGES IN MANUSCRIPT:**
We have clarified the justification for using only these parameters in the Methods section, under the ecomorphology subsection. In the Discussion section, we indicate that the analysis focuses on changes in disparity related to the selected parameters.

3. The focal taxonomic groups are inadequate to support claims about mollusks in general; Claims about mollusks in general should not be based solely on bivalves and gastropods. Even when limited to bivalves and gastropods, only isometric growth is principally considered (related to Major comment 2 above). Given that non-isometric morphological features play important roles in the life modes considered in this study, the claims of this manuscript are likely to be more limited in scope than currently presented.

**Authors’ reply:**
Our focus on bivalves and gastropods is justified because these two groups together account for the vast majority of molluscan diversity (gastropods alone represent roughly 80% of living mollusks), and they are by far the most abundant and informative groups in the mollusk fossil record owing to their high preservation potential. In contrast, groups such as scaphopods or monoplacophorans possess a much more limited and fragmentary fossil record, which presently prevents their reliable inclusion in analyses of morphological disparity. Although cephalopods are a well-studied group from the Late Cretaceous, we were unable to obtain a sufficiently complete and standardized image dataset to incorporate them into the present study. Future studies should aim to incorporate cephalopods once a more complete dataset becomes available.

**CHANGES IN MANUSCRIPT:**
In the Discussion section, we emphasized that the results are limited to the mollusk groups analyzed in this study, namely bivalves and gastropods, avoiding generalizations to all mollusks.

4. Over-discussion
Multiple claims in the manuscript seem over-discussed to me. Given the limited size of the data, the limited sampling and geographic scope, and the limited morphological features analyzed, I recommend that the authors carefully reconsider the claims presented and clarify the scope of what can be reasonably concluded. In paleontological research, the above challenges are difficult to avoid, and I do not believe they diminish the value of the study itself. However, it is crucial to clearly describe what can be claimed based on the data and quantitative analyses. For the value of quantitative analyses using theoretical models, it is important to distinguish between what can be quantitatively supported based on hypotheses and what remains speculative.

**Authors’ reply:**
We agree that in paleontological research, certain limitations are difficult to avoid, as they depend on the fossil records available. In the revised manuscript, we have refined our claims to ensure that they are fully supported by the data and quantitative analyses presented in this study.

**CHANGES IN MANUSCRIPT:**
Throughout the Discussion, we emphasize that the results are limited to the data and quantitative analyses presented in this study.

**Minor comments:**
1. It would be useful for readers to provide specific methods for model parameter estimation. Since this is important for evaluating disparity, also include information on estimation errors if possible.

**Authors’ reply:**
Information on the specific methods used to measure the model parameters is presented in Figure 2. This figure illustrates how the measurements were taken on the specimens and the equations required to obtain the parameters.

2. I recommend adding specimen IDs associated with the database in Table S1.

**Authors’ reply:**
The associated IDs (Catalog number, Institutions, or publication) have been added to the database in Table S1.

**CHANGES IN MANUSCRIPT:**
Supplementary Table S1 has been updated according to the reviewers’ recommendations.

**REVIEWER #3**

We thank you for your valuable comments. Based on your suggestions, we have revised the points related to comparisons with the existing literature in the Discussion section, focused more on the local implications of the results, and improved the presentation of the number of specimens over time.

Introduction:

*Line 34: "morphological studies focused on quantifying the diversity of mollusk shell forms"* Could you add any studies that have this type of study?

**Authors’ reply:**
We have added several relevant references related to the quantification of mollusk shell form diversity.

**CHANGES IN MANUSCRIPT:**
Three new references have been incorporated (cited in line 35).

*Line 40: The authors state that "Various aspects of morphological variability across geological time can be captured by employing diverse disparity measures," but they do not exemplify the changes over geological time. Adding an example in this introductory section would make the ideas clearer.*

**Authors’ reply:**
We have added a sentence to clarify how morphological disparity changes over geological time, highlighting the types of morphological variation observable within morphospace.

**CHANGES IN MANUSCRIPT:**
A clarifying sentence has been added in line 41.

*Lines 48-49: In a very general way, it is mentioned that there are studies with morphospatial approaches in mollusks. However, the authors can provide some examples for this text.*

**Authors’ reply:**
We have included specific examples of morphospace-based approaches applied to mollusks, as cited in this manuscript.

**CHANGES IN MANUSCRIPT:**
A couple of sentences have been added to the end of the second paragraph of the Introduction (line 41).

*Lines 56-60: Taphonomy and diagenesis are absent here; both drastically modify shell morphology and cannot be ignored when designing morphospaces. Could you talk about them? Because they directly impact your results.*

**Authors’ reply:**
We used only the best-preserved specimens to minimize bias from taphonomic and diagenetic deformations. We have also added a more detailed specification of the specimens included in this analysis.

**CHANGES IN MANUSCRIPT:**
A clarification has been added to the last paragraph of the Methodology section.

*Line 62: I understand the "biological perspective" of the extinctions. However the geological perspective can not be just ignorated. Macroevolutionary processes are rarely recognized in the fossil record (small fossil data, taphonomy modifications, taxonomic issues, etc.), and the geological perspective is often mapped only by geochemical data. This is important to be mentioned.*

**Authors’ reply:**
We have added a sentence highlighting the importance of geological perspectives in extinction events.

**CHANGES IN MANUSCRIPT:**
A sentence has been added in line 62.

*Lines 69-71: In fact, paleoenvironments were already being modified throughout the Cretaceous with climate change and faunal turnover. In fact, the Chicxulub impact collapsed niches very quickly, but the collapse of primary production and ocean acidification occurred heterogeneously across the planet. I suggest adding this information so the reader can see the text more clearly.*

**Authors’ reply:**
Thank you for your observation. We have clarified this in the manuscript.

**CHANGES IN MANUSCRIPT:**
A sentence has been added in the manuscript (lines 70–71).

*Lines 69-71 and 75-77: Reference?*

**Authors’ reply:**
We already had references for lines 69–71 and have now added another reference. Lines 75–77 refer to Figure 1, which is cited as a reference [51].

CHANGES IN MANUSCRIPT:
An additional reference has been added in line 71.

*Line 81: The "the most studied macroevolutionary events in the history of life on Earth" is too much. If we are to judge, for life on the planet the Ediacaran-Fortunian boundary was more important.*

**Authors’ reply:**
We used the phrase “is one of the most studied macroevolutionary events” not to imply that it is the most important event but rather to emphasize the extensive amount of research available on this extinction event. However, if this phrasing seems too strong, we can soften it.

**CHANGES IN MANUSCRIPT:**
Accordingly, we have adjusted the phrasing in line 81 to reflect this.

*Line 87: Why were only these classes "Bivalvia and Gastropoda" studied? Any specific reason?*

**Authors’ reply:**
We focused on Bivalvia and Gastropoda, as these groups were best represented in the collections studied. Future studies should aim to incorporate cephalopods once a more complete dataset becomes available.

*Lines 90-96: This information should be in the paper's Discussion.*

**Authors’ reply:**
We appreciate your observation. We have revised these lines to make them more introductory, and the additional content has been moved to the Discussion section.

**CHANGES IN MANUSCRIPT:**
Revisions were made to the last paragraph of the *Introduction* (lines 90–96), and a new paragraph was added to the *Discussion* section (after line 402).

*Figure 1: I suggest including the number of species, genera, and families in each geological age, or presenting the data in a table. A richness scale of 0–800 is too big and difficult to visualize.*

**Authors’ reply:**
We respectfully disagree with the suggestion. Our intention with Figure 1 is to present the overall visual pattern of mollusk diversity in the Eastern Pacific and to highlight the marked taxonomic decline during the Danian. We believe that the current format effectively conveys the major trends in sampled diversity across the late Cretaceous and early Paleogene. Adding species, genera, and family counts to each geological age would detract from this visual overview. For clarity and readability, we prefer to keep the figure as it is.

**Methods:**

Lines 107-110: What was the selection criterion for choosing the families? And why was only one specimen per species studied? Don't you think this might affect your results? Were they all juveniles? Adults? What was the criterion for choosing the specimens? Please provide clear methodological information.

**Authors’ reply:**
We appreciate the reviewer’s comment. We have included a brief justification for this observation in the Methods section.

The families analyzed were selected because they are the most representative of the fossil records of the Eastern Pacific. Data on this fossil record were generated during a large-scale, multi-institutional digitization initiative, for which associated taxonomic resources served as the taxonomic, ecological, and geological (age and locality) foundations for this study. Although intraspecific variation is relevant for understanding morphological diversity, it is generally lower than the variation observed among species, genera, and families. The model used in this study was designed to capture macroevolutionary patterns in overall shell morphology; therefore, small intraspecific differences may not be reliably detected using this model. We selected the best-preserved and most complete fossils available, prioritizing specimens from type collections (holotypes, paratypes, or hypotypes) to ensure accurate representation of each species. Most type specimens correspond to adult ontogenetic stages, which provide the most diagnostic features for species identification.

**CHANGES IN MANUSCRIPT:**
A new paragraph has been added in line 109 (*Methods Section*, *Fossil Material* subsection).

Line 117: In the "Measures and Theoretical Morphospace" section, I suggest adding a paragraph presenting the ontogeny of these bivalve and gastropod mollusks. Throughout the growth process of both, the morphology of the shells changes. Indicating which ontogenetic phase you are working with is important, thus reducing potential data bias.

**Authors’ reply:**
Ontogenetic studies using theoretical morphospaces are particularly interesting because they allow tracking the migration of an individual through morphospace during ontogenetic development (Klug et al., 2016, with ammonoids). However, this was not the objective of the present study. Determining the ontogenetic stage of each bivalve or gastropod species is challenging, and this was not the focus of our study. Nevertheless, specimens from type collections (holotypes, paratypes, or hypotypes) were selected to ensure accurate representation of each species. These type specimens are typically adults, as this stage allows for clearer taxonomic description. Among these, we selected the best-preserved and most complete fossils for our analysis.

**CHANGES IN MANUSCRIPT:**
A new paragraph has been added in line 109 (*Methods Section*, *Fossil Material* subsection).

Lines 144-153: Reference?

**Authors’ reply:**
The first paragraph of the *Ecomorphology* section has now been referenced according to the information in the EPICC Database. We appreciate your observation.

**CHANGES IN MANUSCRIPT:**
A reference has been added to the line 146.

Line 164: In the "Disparity measurements" section. Were normality tests performed on the data? Are they applicable in this case?

**Authors’ reply:**
We appreciate the reviewer’s comment. However, normality tests were not performed because the disparity metrics (Convex Hull Area and Mean Pairwise Distance) are geometric and descriptive measures of morphospace occupation rather than parametric statistical variables. Therefore, the normality assumption is not applicable in this context.

**Results:**

Line 191: In "However, upon closer examination according to the ecological category" when was this "closer examination" performed, was any other test applied? It's important to clarify. Perhaps a Cluster Analysis of the results can help you see how the data clusters over time.

**Authors’ reply:**
Yes, that is correct; this could be confusing. The “closer examination” expression refers to the analysis presented in Figure 5, where the distribution of bivalves is separated according to their ecological categories and time intervals. This differs from Figure 4, in which all bivalves are plotted together without distinction between categories or geological age.

**CHANGES IN MANUSCRIPT:**
The type of “examination” is specified in line 191 of the manuscript.

Lines 195-197: Indeed, for a taxon to persist for 10 million years is a long time, especially under oscillatory paleoenvironmental conditions, such as those at the K-Pg boundary. These paleoecological recoveries may also be a faunal turnover; considering the morphometric replacement of the niches presented here, I think it is important to mention or suggest this possibility.

**Authors’ reply:**
We have clarified this in the manuscript.

**CHANGES IN MANUSCRIPT:**
A clarification regarding the type of paleoecological recovery has been added to line 195.

Line 206, 210, 212, 215, 256: Cite the “species richness” here as well.

**Authors’ reply:**
We have added the number of species in the text so that readers can easily identify them without consulting the corresponding figure.

**CHANGES IN MANUSCRIPT:**
The number of species is specified in lines 206, 212, 215, and 256 of the manuscript.

Lines 209-210: "Immediate" recovery in the fossil/geological record is very difficult to achieve. If we observe Figure 6, ignoring time 3, a decreasing trend (6-c) of Stationary epifauna and Facultatively mobile semi-infauna is notable; however, Facultatively mobile infauna are maintained.

**Authors’ reply:**
We agree that the term “immediate recovery” is not appropriate when referring to geological timescales. Our intention was to emphasize that epifaunal disparity recovered relatively quickly compared with other ecological categories.

**CHANGES IN MANUSCRIPT:**
We have rephrased this sentence to clarify this point and avoid implying an instantaneous recovery (Lines 209–210).

Lines 220-221: Why is the family Mactridae important? References are needed to further support this statement. Is there a reason for the absence of this family in the Paleocene? Taphonomic?

**Authors’ reply:**

The family Mactridae was mentioned because it represents one of the most abundant and diverse bivalve families during the Late Cretaceous and Early Cenozoic, playing an important role in shallow marine environments. However, Mactridae were not recorded in the Paleocene samples analyzed in this study, likely because of the absence of suitably preserved material.

**CHANGES IN MANUSCRIPT:**
Lines 220–221 have been revised accordingly.

Line 227-231: In fact, semi-infaunal facultatively mobile species failed to recover their morphological disparity; quite the opposite. Figure 6 shows a broadening of their morphometric spectrum, in addition to increasing the number of families from one to three. Could this change be a faunal turnover? How did semi-infaunal facultatively mobile species 8achieve such success in the K-Pg? Could this extinction be distinct for different taxa?

**Authors’ reply:**
We believe that there has been a misunderstanding regarding this issue. In Figure 6a (which corresponds to the Paleocene), a decrease in both the number of families and morphospace area is observed. We believe that the confusion arose from how the figure was cited; it has now been clarified to refer specifically to Figure 6a.

**CHANGES IN MANUSCRIPT:**
The caption of Figure 6 has been edited accordingly, and the reference has been corrected in the text (Line 229).

Lines 234-239: The "Facultatively mobile deep infauna" exhibited paleoecological behaviors similar to those of semi-infaunal facultatively mobile species, increasing their diversity and expanding morphospaces. I would like to see an in-depth discussion, considering the forms and other modifications brought about in K-Pg.

**Authors’ reply:**
We agree that the “Facultatively mobile deep infauna” group deserves a more detailed discussion. In the revised text, we clarified that although this group increased in diversity after the extinction event, it did not necessarily result in a broader expansion within the morphospace.

**CHANGES IN MANUSCRIPT:**
A clarification has been added in line 236 to address this issue.

Line 244: Could these disparate values be due to taphonomic and diagenetic factors? If not, how were they differentiated?

**Authors’ reply:**
We believe there may have been a misunderstanding regarding the term “disparate values.” We refer to “disparity values” in the context of disparity measures, not to values that are inconsistent or irregular. Thank you for this suggestion. We have revised the text for improved clarity as suggested. Additionally, in this study, potential biases were minimized by using well-preserved specimens from the standardized stratigraphic sections.

**CHANGES IN MANUSCRIPT:**
*We have improved the wording in line 244 to avoid confusion with the term “disparity.”*

Line 274: What is the number of species?

**Authors’ reply:**
We have added the number of species in the text so that readers can easily identify them without consulting the corresponding figure. We have also specified this information for creeping epifauna (Line 256).

**CHANGES IN MANUSCRIPT:**
The number of species is specified in lines 256 and 274 of the manuscript.

Line 285-286: Suggesting that "these organisms can maintain their morphological diversity through extinction events" without other data is a bit hasty. Is there any other information or comparison that this has occurred over geological time?

**Authors’ reply:**

We agree that this sentence can be interpreted as a broad generalization. Our intention was to highlight that *Turritellidae* exhibit remarkable morphological stability compared with that of other ecological groups.

**CHANGES IN MANUSCRIPT:**
We have refined the writing in lines 285–286 to avoid such generalization.

**Discussion:**

Lina 291: "especially when compared to the prevalence of studies utilizing PCA morphospaces" without presenting and comparing the data is irrelevant. I suggest using the data to demonstrate the weaknesses of the PCA test. Apply PCA tests to your data to show and compare test problems.

**Authors’ reply:**
We agree that mentioning a comparison with PCA-based morphospaces without presenting specific data could be considered irrelevant. Our intention was to emphasize the limited use of theoretical morphospaces compared with more common empirical approaches. However, a formal comparison would require additional methodological details and new graphical analyses, which are beyond the scope of this study but will be addressed in future studies. The sentence has been modified accordingly to avoid referring to methods that were not applied in this study.

**CHANGES IN MANUSCRIPT:**
The first paragraph of the Discussion has been revised to address the reviewer’s comments (Lines 289–295).

Line 296: Was the extinction event at the K-Pg boundary homogeneous and synchronous worldwide? If so, focus on your area of study and discuss it briefly. If not, discuss how it occurred differently in different locations around the world. This would make your discussion more interesting for the reader.

**Authors’ reply:**
Following the previous comment regarding the Introduction, we have now included additional information emphasizing that the effects of the K–Pg extinction occurred heterogeneously across the planet, reflecting spatially and temporally variable conditions. Although the global nature of this event is well documented, marine records from the western Pacific coast of North America remain relatively scarce. To address this, we incorporated new discussion paragraphs highlighting examples of marine environmental changes observed in nearby and other regions of the world.

**CHANGES IN MANUSCRIPT:**
A new paragraph discussing the heterogeneous effects of the K–Pg extinction has been added in the “Ecomorphology across the K–Pg boundary” section (between lines 370 and 371).
Additionally, the paragraph originally located at lines 382–386 has been repositioned after the second paragraph of this subsection (after line 365).

Lines 309-312: This paragraph seems more like a result.

**Authors’ reply:**
We have revised the paragraph to improve the clarity of the text and remove any phrasing that could be interpreted as a description of the results of the study.

**CHANGES IN MANUSCRIPT:**
The text in paragraphs 309-312 has been rephrased.

Lines 317-318: "Further exploration of proper parameter detection could contribute to the development of new ecomorphospaces for Helicoidal", please clarify the reason that prevented you from carrying out this “exploration of proper parameter detection”. I understand that ecology has a direct impact, but it needs to be better supported.

**Authors’ reply:**
We agree that this information is not essential to the study. The last sentence of the paragraph has been revised to improve the discussion.

**CHANGES IN MANUSCRIPT:**
The final part of the paragraph (lines 317–318) has been removed, and a new sentence has been added.

Lines 335-337: Result?

**Authors’ reply:**
We agree that the information in these lines has been revised to better fit the Discussion section.

**CHANGES IN MANUSCRIPT:**
Lines 335–338 have been modified accordingly.

Lines 357-359: In fact, as a reader, the results and discussion present only information/data from morphospaces. I suggest that you develop an in-depth discussion, including paleoenvironmental changes and sedimentary processes, as they directly impact the ecology of animals.

**Authors’ reply:**
Following this suggestion, we incorporated additional information and a more detailed discussion of the paleoenvironmental context that may explain the observed patterns of morphospace occupation and recovery.

**CHANGES IN MANUSCRIPT:**
New complementary information has been added after lines 355 and 381.

Lines 388-389: The "varied tectonic activity due to ongoing subduction" hadn't been mentioned before. Are they relevant to your results? If so, it's important to discuss this throughout the discussion, not just here. Be careful with "generally maintained an oceanic aspect" as you previously stated in line 367 the "Acidification and suppression of primary productivity in oceans". Make it clear what type of oceanic maintenance you're referring to.

**Authors’ reply:**
It was out of context to discuss the geology of the area without introducing it. We included this information to clarify that the region was a warm-water ocean not influenced by the transgressive and regressive processes of an epicontinental sea. We also revised the phrase “generally maintained an oceanic aspect” to specify that it refers to the persistence of marine conditions, rather than stable oceanic productivity. The text has been modified accordingly to ensure consistency with the rest of the discussion.

**CHANGES IN MANUSCRIPT:**
The discussion paragraph covering lines 387–392 has been revised, and additional information has been included in the introduction (line 89) to improve coherence. In addition, the lines 395-397 have been modified.

Lines 396-397: The authors said "the broad geographic distribution of the fossils studied here", but they did not clearly present how many were studied from each locality, age, each genus, species, etc. This data is in the supplementary table, but I suggest presenting another reduced table in the results section to facilitate visualization of the data.

**Authors’ reply:**
We agree that analyzing morphological changes in relation to specific localities, age, and stratigraphic context provides valuable insights. However, we believe that such an analysis would be better suited for a separate study focused more precisely on the geological and stratigraphic aspects. The current study aimed to provide a broader overview of morphological changes within morphospaces across relatively large temporal scales. This approach will be considered in future research.
