# Supplementary material for: Morphological disparity across the K-Pg boundary in mollusk shells: A theoretical morphology approach
Source: PLoS One. 2026 Feb 20;21(2):e0343127. doi: 10.1371/journal.pone.0343127 (PMC12923056; doi:10.1371/journal.pone.0343127)
Supplement: Attachment — Submitted filename: Response_to_Reviewers-R2.docx [file pone.0343127.s005.docx]

**RESPONSE TO THE REVIEWER**

Manuscript: **PONE-D-25-43258**

Morphological disparity across the K-Pg boundary in mollusk shells: a theoretical morphology approach

First, we would like to thank the reviewer for their careful reading of the manuscript.

**Reviewers’ comments are in blue italics, authors’ replies are in plain text, and changes in the manuscript considering the recommendations are underlined. In the revised version of the manuscript, the changes have been highlighted in orange.**

**REVIEWER #1**

*1. The corresponding author listed in the manuscript does not match the author whose e-mail is provided in the article. Please check.*

**Authors’ reply:**
It was corrected.

**CHANGES IN MANUSCRIPT:**
The corresponding author is indicated with an asterisk.

*2. Line 267: the cited figure for the MPD display appears to be Fig. 8c, not 8b. If it's the case, please verify and correct figure citations throughout the text.*

**Authors’ reply:**
The correct figure is Figure 8c. We appreciate your observation.

**CHANGES IN MANUSCRIPT:**
The figure citation has been corrected to Fig 8c in line 267.

*3. In Fig. 8c the green line representing “Facultatively mobile semi-infauna” appears detached from its marker in Time 3. Is this intended? This ends up affecting the statement in l. 263-264 and l. 375: “(…) each category showed a notable decrease during the epoch immediately following the mass extinction (…)”, which wouldn’t be necessarily the case of facultatively mobile semi-infaunal gastropods.*

**Authors’ reply:**
You are right. The green line appears to be detached from its marker. Facultatively mobile semi-infaunal gastropods, like the other ecological categories, showed a decrease immediately following the mass extinction event.

**CHANGES IN MANUSCRIPT:**
Figure 8b has been corrected and replaced.

*4. Line 296: please check whether the panel showing “helicoid shells with narrow apertures” should be Fig. 8c.*

**Authors’ reply:**
Thank you for your observation; the correct panel in Figure 8 is “b”.

**CHANGES IN MANUSCRIPT:**
The figure citation has been corrected to Fig 8b in line 296.

*5. Line 317 states that disparity measures recorded a loss of morphological diversity in all groups, associated with the mass extinction event. Is this also true for the Facultatively mobile infaunal bivalves?*

**Authors’ reply:**
This is also true for facultatively mobile infaunal bivalves when the Convex Hull Area is measured, indicating a reduction in morphological variation following the mass extinction event.

*6. In the legend of Figure 5, “Stationary epifauna” text is displayed with a different font than the rest of the figure.*

**Authors’ reply:**
We have made the requested changes.

**CHANGES IN MANUSCRIPT:**
The legend of Figure 5 has been modified to use consistent font.

*7. Lines 342-344: This paragraph was a great addition to the manuscript, only this specific sentence about ornamentation seems a bit textually disconnected. Please consider rewording or removing that sentence for better flow.*

**Authors’ reply:**
We have added a sentence to improve the coherence of this paragraph.

**CHANGES IN MANUSCRIPT:**
A sentence has been added in line 342-344.

*8. Lines 357–361 repeat the claim that infaunal and semi-infaunal taxa display less morphological variation due to environmental constraints. Please combine or remove the redundancy.*

**Authors’ reply:**
We have avoided redundancy in the paragraph to improve the flow of this part of the discussion.

**CHANGES IN MANUSCRIPT:**
The sentence in lines 358–360 has been deleted, and a small comment has been added in lines 357-358.

*9. Line 378-380 and 416: By looking at the presented data, it seems that facultatively mobile infaunal bivalves’ occupied morphospace area was able to return to its pre-extinction state.*

**Authors’ reply:**
The occupied morphospace of facultatively mobile infaunal bivalves remained nearly stable. However, the lines in question (378-380 and 416) refer to the general morphospace of the Bivalvia and Gastropoda classes.

**CHANGES IN MANUSCRIPT:**
A clarification specifying “Bivalvia and Gastropoda” has been added to line 414.

*10. Line 386: “Inoceramidae” (typo)*

**Authors’ reply:**
Thank you for your observation.

**CHANGES IN MANUSCRIPT:**
The typographical error in line 384 has been corrected in the revised manuscript.

*11. Lines 425-426: The word “here” is used twice in the sentence; one could be suppressed.*

**Authors’ reply:**
Thank you for your observation.

**CHANGES IN MANUSCRIPT:**
The duplicate word has been removed from the text.

*12. The ecological interpretation that burrowing and mobility were advantageous post-extinction is compelling. Consider briefly linking this to modern analogues or experimental observations that support the advantage of mobility/burrowing under environmental stress (one or two citations would strengthen the argument.*

**Authors’ reply:**
In the Discussion section, we have already cited several references to previous studies documenting molluscan responses to marine environmental stress. These references focus on extinction events and are therefore more appropriate for the paleoenvironmental aims of the present study.

*13. Throughout the text MPD is called Mean Pairwise Distance, but figure captions read “Main Pairwise Distance.” Please correct captions to Mean Pairwise Distance for consistency.*

**Authors’ reply:**
Thank you for these observations.

**CHANGES IN MANUSCRIPT:**
Figure captions were corrected (Figures 5 and 8)

*14. Check references and figure captions for minor typos and formatting. Otherwise, the manuscript appears well proofread.*

**Authors’ reply:**
We have checked the references and figures captions for typographical or formatting mistakes.

**CHANGES IN MANUSCRIPT:**
Formatting corrections were made to homogenize the references. No changes are required in the figure captions with respect to typographical or formatting mistakes.
